# Associative learning of non-sugar nectar components: amino acids modify nectar preference in a hawkmoth

**DOI:** 10.1242/jeb.234633

**Published:** 2021-06-18

**Authors:** Geoffrey T. Broadhead, Robert A. Raguso

**Affiliations:** Department of Neurobiology and Behavior, Cornell University, Ithaca, NY 14853, USA

**Keywords:** *Manduca*, Pollinator, Nutrition, Foraging behavior, Color, Fitness

## Abstract

The nearly ubiquitous presence of amino acids in the nectar of flowering plants has led to significant interest in the relevance of these compounds to pollinator behavior and physiology. A number of flower-visiting animals exhibit behavioral preferences for nectar solutions containing amino acids, but these preferences vary by species and are often context or condition dependent. Furthermore, the relative strength of these preferences and potential influence on the foraging behavior of flower-visiting animals remains unclear. Here, we used innate preference tests and associative learning paradigms to examine the nectar preferences of the flower-visiting hawkmoth *Manduca sexta*, in relation to both sugar and amino acid content. *Manduca sexta* exhibited a strong preference for higher sucrose concentrations, while the effect of amino acids on innate feeding preference was only marginally significant. However, with experience, moths were able to learn nectar composition and flower color associations and to forage preferentially (against innate color preference) for nectar with a realistic amino acid composition. Foraging moths responding to learned color cues of nectar amino acid content exhibited a behavioral preference comparable to that observed in response to a 5% difference in nectar sucrose concentration. These results demonstrate that experienced foragers may assess nectar amino acid content in addition to nectar sugar content and caloric value during nectar-foraging bouts.

## Abstract (precis)

**Summary:** Experienced hawkmoths prefer floral nectars containing amino acids and are able to learn floral cues associated with nectar composition in order to preferentially visit amino acid-containing flowers.

## INTRODUCTION

Foraging animals are routinely faced with a variety of decisions, the outcomes of which can directly influence an individual's condition and reproductive fitness (Behmer, 2009). Flower-visiting insects, in particular, make hundreds of decisions within a given day, including choosing between foraging patches and discriminating between flowers of variable reward quality, while simultaneously balancing nutrient intake and metabolic demand (Heinrich, 1976; Watt et al., 1974). Traditionally, these foraging decisions have been investigated with an emphasis on net energy gain (Petanidou and Vokou, 1990; Roubik et al., 1995; Cnaani et al., 2006). More recently, taste perception and macronutrient ratios of pollen have also been shown to play a role in influencing flower choice in foraging bees (Muth et al., 2016; Vaudo et al., 2016; Ruedenauer et al., 2019), demonstrating a more balanced regulation of nutrient intake similar to that described in other insect herbivores (Behmer, 2009). Distinct from the taste and nutritional value of pollen, floral nectar itself is a complex biological solution containing a myriad of proteins, lipids, free amino acids, inorganic ions and secondary metabolites in addition to sugar (Afik et al., 2006; Kessler et al., 2012; Nicolson and Thornburg, 2007). Yet, while the effect of nectar toxins and other distasteful compounds on foraging behavior has been investigated in a variety of contexts (Adler, 2000; Stevenson et al., 2017), the influence of non-sugar nectar nutrients has received less attention. This is likely a result of the availability of more concentrated nutritional rewards available on the same flower (i.e. pollen), diminishing the presumed relevance of more dilute nectar solutes. In the case of flower-visiting Lepidoptera and other specialized nectarivores, however, nectar macronutrients are likely to have increased relevance because of the specialized morphology of the proboscis, which typically precludes the consumption of solid/non-nectar resources, and the nitrogen- or protein-limited state of many adult Lepidoptera, which are otherwise restricted to resources stored during larval development (Boggs and Ross, 1993; O'Brien et al., 2002; Mevi-Schütz and Erhardt, 2005).

Sugars are typically the dominant component of floral nectar and have been the focus of extensive behavioral and ecological research, yet the presence of nitrogenous compounds in nectar has been documented since the mid-20th century (Ziegler, 1956; Lüttge, 1961). This category of nitrogenous nectar metabolites includes nectar alkaloids and proteins as well as numerous protein-forming and non-protein-forming free amino acids (Adler, 2000; Beutler, 1935; Baker and Baker, 1973; Nicolson and Thornburg, 2007). Although the composition and concentration of nectar amino acids varies both within and between plant species, their presence at approximately millimolar levels is considered nearly ubiquitous in flowering plants (Baker and Baker, 1973; Gardener and Gillman, 2001; Petanidou et al., 2006) and behavioral preferences for amino acids have been documented in foraging animals across a variety of taxa including flies, ants, bees and butterflies (Alm et al., 1990; Erhardt and Rusterholz, 1998; González-Teuber and Heil, 2009; Lanza, 1988; Rathman et al., 1990).

Despite the frequently documented and widespread preferences for free amino acids, insight into the broader ecological and behavioral relevance of nectar amino acids remains limited. Most studies investigating preferences for nectars containing amino acids are conducted using equimolar sugar concentrations, thus experimentally isolating the effect of amino acids on nectar preference (Alm et al., 1990; Erhardt, 1991; Erhardt and Rusterholz, 1998). This approach is successful in targeting amino acids specifically and provides valuable foundational evidence but does not address how well a statistically significant preference might translate to a broader context in which natural nectars exhibit multiple axes of variation. For example, the sugar concentration of a natural floral nectar may fall within a relatively consistent range for a given species or group of species with shared pollinators. However, nectar sugar concentration responds to numerous biotic and abiotic factors and significant variation is well documented between individual flowers (same plant), different plants, populations and species, especially as a result of the presence of floral yeasts (Canto et al., 2008; Herrera et al., 2008). To consider preferences for amino acids or other less abundant nectar compounds as relevant factors influencing pollinator foraging in a wider context, that statistical preference should withstand some amount of variation in other nectar components (i.e. sugar concentration). There is some evidence in nectivorous bats that nectar amino acids modify foraging behavior and interfere with discrimination between nectars of varying sugar concentrations (Rodríguez-Peña et al., 2013); however, most experimental tests addressing the influence of amino acids are restricted to comparisons involving a single standardized sugar concentration.

Furthermore, behavioral tests of nectar preferences compel a variety of important considerations, one of which is inherent foraging differences between species and, potentially, between sexes of the same species. For example, the foraging decisions of a solitary forager are influenced by individual experience, nutritional status and that individual's own energy budget. A collectively foraging species, such as the honeybee, often employs specialized foragers (workers) which benefit from shared information and resources within the hive, and have optimized acceptance thresholds for specific foraging tasks (i.e. nectar or pollen collection) (Değirmenci et al., 2018; Detrain and Deneubourg, 2008; Farina et al., 2007; Lihoreau et al., 2015; Pankiw et al., 2004; Seeley, 1989). Thus, a single forager from a collectively foraging species may exhibit strong, narrow preferences for specific nectar chemistry while a solitary forager under the same conditions may be forced either to compromise and balance multiple nutritional demands simultaneously, or to consume large quantities of a less preferred nectar before a higher quality resource is located or specific nectar associations are learned (Lihoreau et al., 2017; Persson, 1985; Tepedino and Parker, 1982). Additionally, in nectar preference tests, the composition of the artificial nectar used can have a strong influence on the experimental outcome. There is evidence for associations between non-sugar nectar chemistry and pollinator identity (Baker and Baker, 1983; Baker et al., 1998; Kaczorowski et al., 2005; Stiles and Freeman, 1993), and behavioral responses to single amino acids or amino acid blends at the correct concentration but arbitrary ratios can vary dramatically from responses to more natural nectar blends (Blüthgen and Fiedler, 2004; González-Teuber and Heil, 2009; Shiraishi and Kuwabara, 1970).

In this study, we used virgin *Manduca sexta* (Lepidoptera: Sphingidae) hawkmoths of both sexes in the absence of any prior nutritional stress to determine whether foraging moths exhibited any innate preference for nectars containing realistic concentrations of amino acids. We further examined the relative strength of that preference within the context of known preferences for high-sucrose nectars, if the presence of amino acids in nectar was sufficient to modify foraging decisions away from those predicted by sugar concentration alone. Finally, using experimental foraging arrays, we examined whether experienced foragers were able to associate floral cues with nectar amino acid content and forage selectively in a way that might mitigate nitrogen limitation.

## MATERIALS AND METHODS

### Animal care

Adult *Manduca sexta* (Linnaeus 1763) moths used in behavioral trials were the offspring of animals acquired from a laboratory colony maintained at the University of Arizona, Tucson, AZ, USA (Stillwell and Davidowitz, 2010). Developing larvae were fed *ad libitum* on an artificial diet adapted for *M. sexta* (Bell and Joachim, 1976) and modified by Goyret et al. (2009), by substituting cornmeal for wheat germ, as a more appropriate source of visual pigment precursors. Moths were maintained at 24°C on a 16 h:8 h light:dark photoperiod in a humidified (ca. 50%) incubator. At the wandering stage, larvae were transferred to wooden pupation boxes (Yamamoto, 1969). Pupae nearing eclosion were sorted by sex and transferred to separate 33 cm×33 cm×60 cm screen cages (BioQuip, Inc., Rancho Dominguez, CA, USA), maintained at 24°C with a consistent 16 h:8 h light:dark photoperiod. Upon eclosion, newly emerged adult moths were maintained in the same way for 3–4 days, unfed, prior to the start of behavioral trials. All behavioral trials were conducted during the initial 120 min of scotophase, as this time frame occurs within a peak in activity for *M. sexta* (Sasaki and Riddiford, 1984; Broadhead et al., 2017). Moths of both sexes remained unmated for the duration of all experiments.

### Artificial nectar solutions and nectar amino acid measurements

To determine ecologically realistic amino acid concentrations for artificial nectar solutions, we harvested nectar from individual flowers of the yellow evening primrose *Oenothera flava* subsp*. taraxacoides* (Onagraceae). These flowers are hawkmoth pollinated (Gregory, 1964), and produce large volumes of nectar (ranging from 6.2 to 56 μl standing crop per flower) sufficient for chemical analyses (Raguso et al., 2007). We germinated seeds of *O. flava* subsp. *taraxacoides* (for accession information, see Summers et al., 2015) and transplanted seedlings into 6 inch pots containing a 60/40 mix of Sun-Gro Metro-Mix 360/Perlite, which were watered daily. The plants were grown in common-garden greenhouse conditions with day/night temperatures at 24°C/21°C and a 12 h photoperiod. Flowering began approximately 6–10 weeks after germination, and nectar samples were collected within 1 h of a flower's opening and stored at −80°C until analysis. Nectar volumes were sufficient for analysis of individual, rather than pooled, nectar samples (*n*=52).

Nectar amino acids were measured using the AccQ-Tag protocol (Waters Corp., Milford, MA, USA), which has been previously used for nectar amino acid analysis (Gardener and Gillman, 2001). Sample processing followed that of Macdonald et al. (2012). Briefly, individual nectar samples were filtered using 0.45 μm centrifuge filter (Corning, Inc., Corning, NY, USA) by centrifugation at 1500 ***g*** for 10 min, and 2.5 µl filtrate was derivatized with AccQ-Tag, following the manufacturer's protocol, and injected into a Waters Acquity UPLC with PDA detector and AccQ-Tag Ultra 2.1×100 mm column. The gradient was: 0–0.54 min, 99.9% A and 0.1% B; 0.54–5.74 min, 90.9% A and 9.1% B; 5.74–7.74 min, 78.8% A and 21.2% B; 7.74–8.04 min, 40.4% A and 59.6% B; 8.04–8.64 min, 10% A and 90% B; 8.64–8.73 min 99.9% A and 0.1% B; 8.73–9.50 min, 99.9% A and 0.1% B (linear between each time point), where A is 90% AccQ-Taq Ultra Eluent A in water, and B is AccQ-Tag Ultra Eluent B. Amino acids were determined by retention time comparison to standards, 1, 25, 50 and 100 pmol and protein-amino acids μl^−1^ (Waters amino acid hydrolysate standard #088122, supplemented with asparagine, tryptophan and glutamine).

Artificial nectar solutions containing l-amino acids were based on the mean concentrations of individual amino acids in these nectar samples (Table 1), using pure standards obtained from Sigma-Aldrich Corp. (St Louis, MO, USA). The standardized sucrose level of 25% d-sucrose (w/v) was chosen to match the mean natural sucrose level in floral nectar of *O. flava* (Raguso et al., 2007).

**Table 1.**
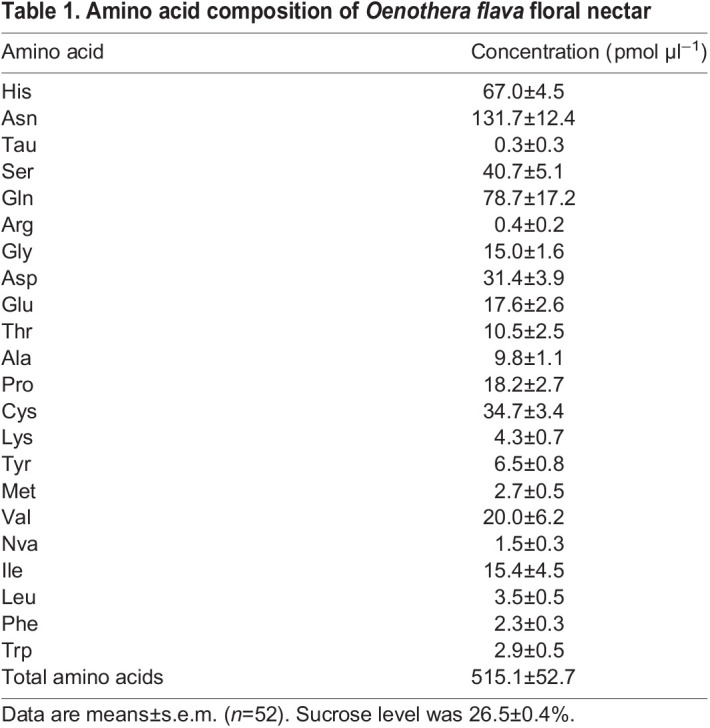
Amino acid composition of *Oenothera flava* floral nectar

### Innate nectar preference

Innate preference tests consisted of 2-choice trials, in which individual naive *M. sexta* were presented with two artificial flowers, each containing a different nectar solution, and nectar consumption was used to determine preference. The experimental arena consisted of a 3 m×1 m×1 m laminar flow flight tunnel, maintained at an illuminance of 8.79±0.14 lx with an airflow of 1.2 m s^−1^. Artificial flowers used in these experiments were constructed from 3 cm diameter plastic funnels (either blue or white) inserted into a microcentrifuge tube serving as a nectar reservoir. The 2-choice array was placed at the upwind end of the flight tunnel with the artificial flowers mounted at a 45 deg angle 40 cm above the floor, and separated laterally by 25 cm. A cotton swab was placed immediately upwind of each artificial flower and treated with one drop of bergamot essential oil, which is chemically similar to many hawkmoth-pollinated flowers and is a reliable feeding attractant for *M. sexta* (Goyret and Raguso, 2006).

These 2-choice trials were conducted in two stages: an initial pre-trial exposure followed by the experimental trial when preferences were measured. Pre-trial exposures were necessary in this experiment to ensure that all moths encountered and tasted both experimental nectar solutions before preference was measured. This pre-trial phase began when a single naive moth was introduced at the downwind end of the flight tunnel and a pair of artificial flowers (blue and white), each augmented with 25 μl of different nectar solutions, was introduced upwind. After initiating flight, individual moths were given 5 min to begin foraging from the artificial flowers. Moths that did not forage or did not successfully consume all nectar from both pre-trial flowers (50 µl in total) were eliminated from the experiment. After experiencing both nectar options during this pre-trial exposure, moths were returned to the downwind end of the flight tunnel and re-released to forage on newly refilled artificial flowers now containing 2 ml of the same nectar solutions. Flower location and nectar–color pairings were held constant for individual moths but were randomized between animals so that each location and color–nectar pairing was equally represented in the data. There were no moths that failed to forage in the experimental trial after successfully completing the pre-trial phase of the experiment. Trials were not restricted to a time limit and moths were allowed to feed until satiety, at which point they stopped flying (generally <10 min of active foraging). At the end of each trial, the volume of nectar consumed from each of the two artificial flowers was measured and a preference index (PI) was calculated based on consumption. Trials were designed such that experimental nectar solutions were always tested against a standard control nectar solution consisting of 25% sucrose (w/v), and the PI was calculated as follows:(1)
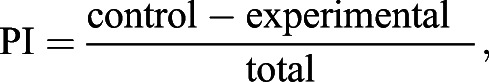
where ‘control’ refers to the consumed volume (µl) of the 25% sucrose solution, ‘experimental’ refers to the consumed volume (µl) of the experimental nectar solution, and ‘total’ refers to the total consumed volume (µl). Positive values of PI approaching +1 indicate a preference for the standard 25% sucrose solution while PI values approaching −1 show preference for the experimental nectar, and PI=0 represents no preference between the two. Nectar solutions compared against the 25% sucrose standard consisted of 5%, 10%, 15%, 20% and 25% sucrose (w/v) solutions. An additional 5 treatments consisted of these same sucrose concentrations with the addition of realistic levels of nectar amino acids (Table 1), for a total of 10 nectar treatments (Table 2). A minimum of 20 moths was tested for each experimental nectar solution (*N*=203) and all trials were conducted in a randomized order.

**Table 2.**
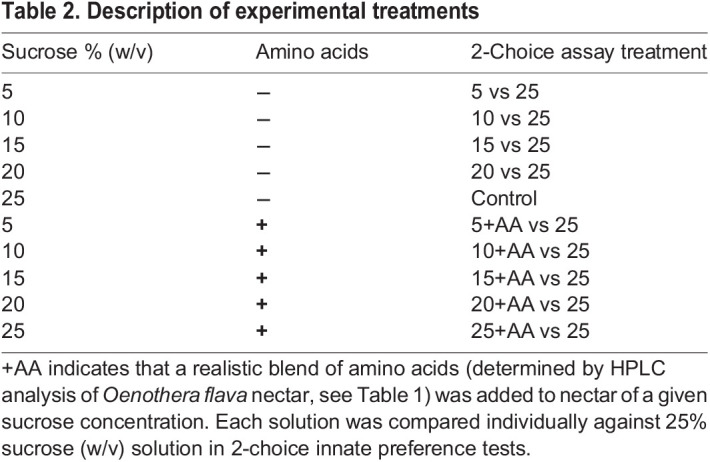
Description of experimental treatments

### Associative learning

**Fig. 1.**
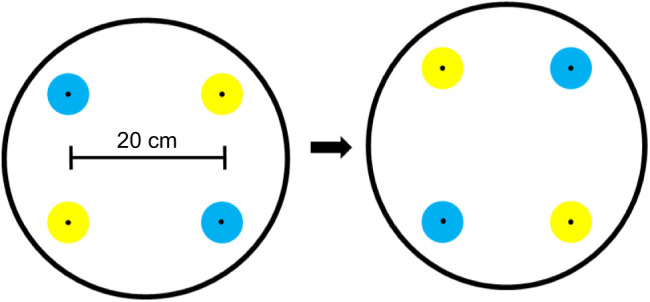
**Foraging array.** The foraging array consisted of a rotating disc with four artificial (blue/yellow) flowers positioned 20 cm apart. This was rotated 90 deg after every three visits from foraging moths to avoid positional learning.

**Fig. 2.**
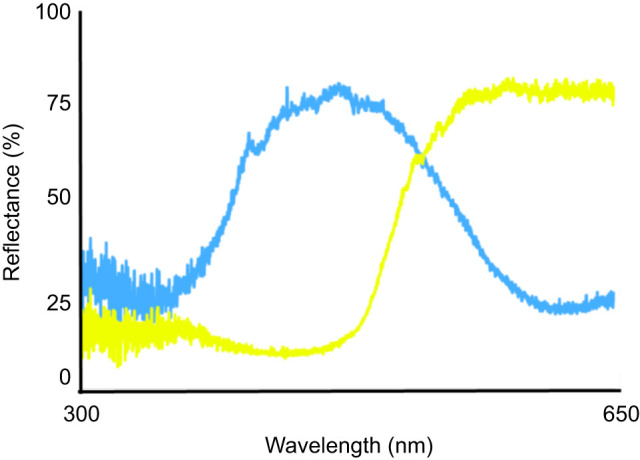
**Average reflectance spectra of the artificial paper flowers.** *n*=6 for each color.

The ability of foraging moths to associate floral cues with nectar amino acid content was assessed using a flower color–nectar composition associative learning paradigm modeled after previous studies of another hawkmoth species, *Macroglossum stellatarum* (Kelber, 1996). The experimental arena consisted of a 142 cm×61 cm×61 cm (LWH) screen cage (BioQuip, Inc., Rancho Dominguez, CA, USA), with an illuminance of 10 lx. A foraging array consisting of a rotating plastic disc with spaces for four artificial flowers mounted 20 cm apart (each space scented uniformly with bergamot oil as described in the previous experiment) was placed at one end (Fig. 1). Artificial flowers were constructed from paper discs folded into 3 cm diameter paper funnels and attached to 100 μl plastic pipette tips, which were sealed at one end and served as nectar reservoirs. In this experiment, the color associations used were blue or yellow flowers, which were constructed from Astrobrights^®^ paper in the colors ‘Lunar Blue’ and ‘Solar Yellow’, respectively (Neenah Paper, Inc., Alpharetta, GA, USA). These papers were chosen because the colors differed in wavelength but were similar in reflectance intensity as measured using an Ocean Optics USB4000 miniature fiber optic spectrophotometer with a deuterium–tungsten lamp and a fiber optic probe to provide standard illumination (Fig. 2). Measurements were taken with the probe mounted at a 45 deg angle and placed directly over the paper flower against a black fabric background and shielded from ambient light. Spectral data were calibrated against a white diffuse reflectance standard (Ocean PN WS-1) set to 100%. Measurements were taken every 0.2 nm, from 300 nm (UV) to 650 nm (red) wavelengths (Fig. 2).

Beginning with naive animals, individual moths were introduced to the end of the experimental arena opposite the artificial flower array. Similar to the protocol used in innate preference tests, moths were allowed 5 min after taking flight to begin foraging. Moths were initially presented with only two flowers (one blue, one yellow), each containing 20 μl of a different nectar solution. As the moth began foraging, first choice (determined by extending the proboscis and probing the flower) was recorded, and the moth was monitored until it was observed to have fed from both flower types. Moths that did not feed from both flower types, thereby encountering both nectar choices, were excluded from the experiment.

Following this initial stage, the foraging array was refilled with two new flowers of each type in alternating positions (four flowers total; Fig. 1), with each flower containing 20 μl of the appropriate nectar solution. At this stage, moths were allowed to forage until satiety. Artificial flowers were refilled after each visit and the entire array was rotated clockwise 90 deg after every third visit to prevent positional learning and ensure learned associations between flower color and nectar type (similar to Kelber, 1996). At the end of each trial, the moth's total number of visits was recorded and the percentage visitation to each of the two flower types was calculated. This procedure was repeated across three consecutive days. Here, the combination of artificial flowers, deep nectar tubes (to visually obscure the nectar reward), and a uniformly scented rotating array was important to ensure that flower color was the only consistent cue of a flower's nectar composition (as opposed to other visual cues, flower position or differences in floral scent).

As in the innate preference test, the experimental nectars used in this experiment (guided by the results of the previous experiment) were tested against a standard 25% sucrose (w/v) solution. Under these experimental conditions, moths exhibited a strong bias in favor of yellow artificial flowers. Therefore, rather than conducting a fully combinatorial experiment in which each nectar–color combination was tested, moths were instead tested by training them away from their initial color bias by placing the nectar of presumed higher quality in flowers of the less preferred color (blue). This approach was previously used by Balkenius and Kelber (2006) as a high stringency test of the ability of the diurnal hawkmoth *M. stellatarum* to learn against a strong innate preference for blue. Experimental treatments and nectar–color pairings are shown in Table 3.

**Table 3.**
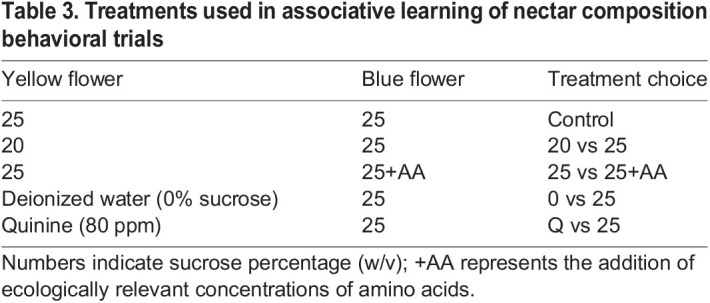
Treatments used in associative learning of nectar composition behavioral trials

### Statistical analyses

All analyses were conducted in R (http://www.R-project.org/). In innate preference tests, side bias (left or right) in the control treatment was evaluated using Student's *t*-test. PIs across all treatments were compared using ANCOVA, with moth sex and the presence of nectar amino acids included as factors and sucrose concentration as a covariate. In associative learning trials, Chi-squared goodness of fit test was used to determine whether first-choice color bias deviated from the expected frequency (50%), and Fisher's exact test was used to evaluate change in first-choice bias over the three trial days. Foraging preferences over time were evaluated using repeated measures ANOVA including trial day as a within-subjects factor, with sex and treatment as between-subjects factors.

## RESULTS

### Innate preference

**Fig. 3.**
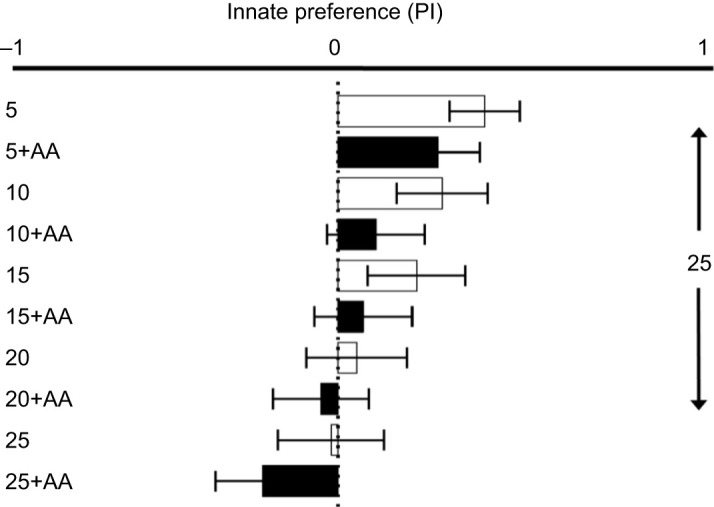
**Innate preference indices of naive *Manducasexta* presented with experimental nectars of varying sucrose or amino acid content, compared with a standardized 25% sucrose control solution.** Preference index (PI) values are shown on a scale of −1 to 1, with 0 indicating no preference. Negative values indicate a preference for the experimental solution, while positive values indicate a preference for the control solution. Experimental treatments containing only sucrose (numbered) are represented by white bars, and black bars represent experimental solutions containing sucrose plus amino acids (AA). Treatment abbreviations correspond to those in Table 2.

In the innate preference tests, moth PI under control conditions (25% sucrose compared against itself) approached zero (Fig. 3; PI=−0.03), and no side bias (left or right placement in the floral array) was detected (Student's *t*-test, *P*=0.35). When analyzed across all treatments, sucrose concentration had a highly significant positive effect on moth nectar preference (*P*<0.0001), as expected. The addition of realistic concentrations of amino acids to experimental nectars showed a trend towards increased preference for amino acid-containing nectar, but this effect was marginal (*N*=203, *P*=0.06) (Table 4). The innate preferences of male and female moths were not significantly different (*P*=0.67), and there was no interaction between sucrose concentration and the presence of amino acids (*P*=0.97) (Table 4).

**Table 4.**
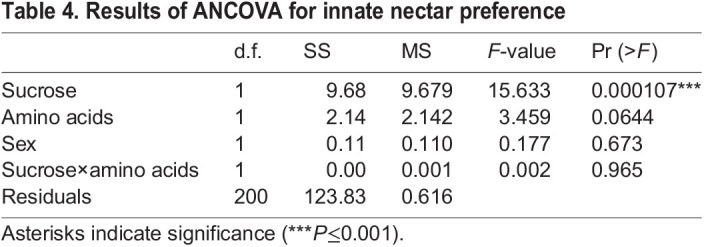
Results of ANCOVA for innate nectar preference

### Associative learning

**Fig. 4.**
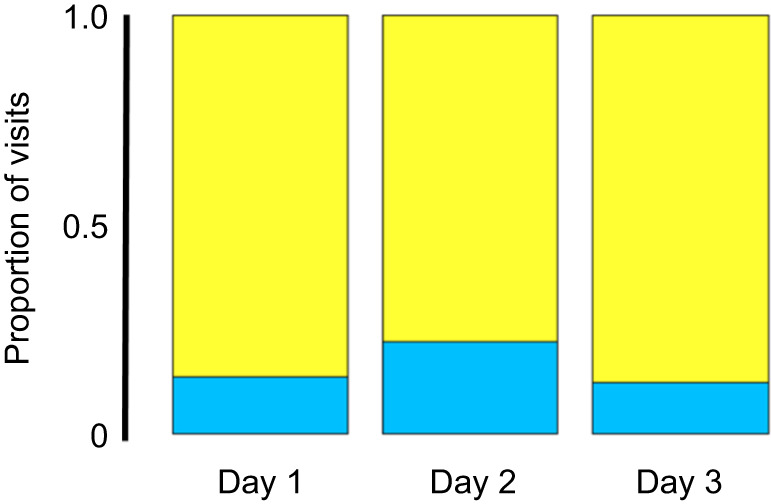
**First-choice bias in first-choice test across 3 trial days.** On day 1, prior to any experience, 86.3% of all first-choice visits were to yellow experimental flowers (χ^2^=38.479, d.f.=1, *P*<0.0001). This first-choice bias did not differ significantly in later trial days (*P*=0.26).

**Fig. 5.**
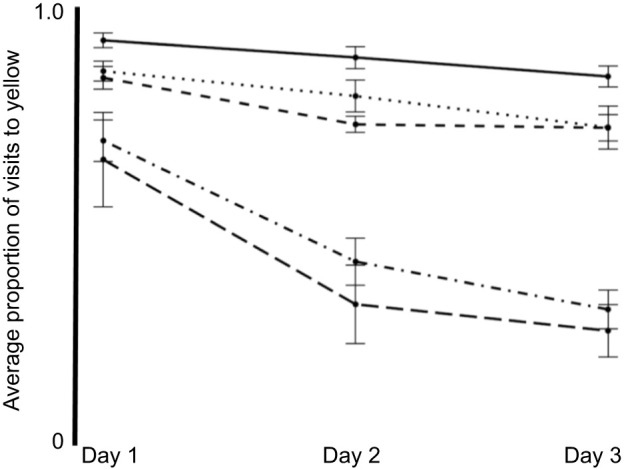
**Color cues of nectar composition alter foraging behavior after learning.** With experience, foraging moths were able to associate floral cues (i.e. color) with nectar composition and lessen the influence of innate biases. Rewarding treatments were: control (solid line), 20 versus 25 (dotted line) and 25 versus 25+AA (dashed line). Non-rewarding or aversive treatments were: 0 versus 25 (dot-dash line) and quinine (Q) versus 25 (long-dash line). Nectar and flower–color pairings are described in Table 3.

Prior to training, the first choice of experimental animals revealed a significant color bias in favor of yellow over blue artificial flowers on first exposure (χ^2^=38.479, d.f.=1, *P*<0.0001) and this first-choice bias remained consistent across all 3 days (*P*=0.26) (Fig. 4). In the control treatment, in which blue and yellow flowers each contained identical 25% sucrose solutions, this strong preference for yellow flowers was maintained across all 3 days of the experiment (Fig. 5). After 3 days of foraging experience, a 3-way repeated measures ANOVA showed that nectar treatment, trial day and the sex of the individual moth were each highly significant factors affecting nectar preference (Table 5). Pairwise comparisons using Tukey's HSD test showed that the 25 versus 25+AA treatment was significantly different from the control (preference for 25+AA, *P*<0.01). Additionally, 20 versus 25 was also significantly different from the control (preference for 25, *P*=0.033). This 5% difference in sucrose concentration (20 versus 25) was not statistically distinguishable from the effect of adding amino acids to otherwise identical sugar solutions (25 versus 25+AA; *P*=0.97). The non-rewarding (0% sucrose) or aversive treatments (80 ppm quinine) were not statistically different from each other (*P*=0.75), but both were statistically different from all other treatments (*P*>0.05 in all pairwise comparisons).

**Table 5.**
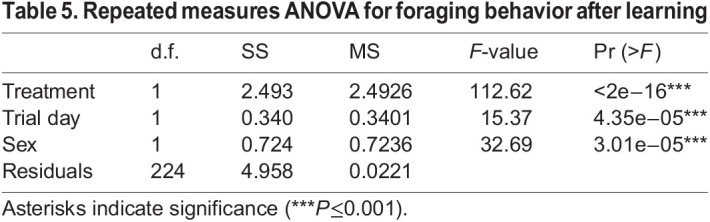
Repeated measures ANOVA for foraging behavior after learning

## DISCUSSION

From a physiological perspective, widespread preferences for nectar amino acids (and amino acids in general) are logical and can serve as a means of replenishing essential, sometimes limiting, nutrients for herbivores and flower-foraging insects (Brodbeck and Strong, 1987; Behmer and Joern, 1993). Nevertheless, there are a number of counter-examples, particularly in the Lepidoptera, that demonstrate either a lack of innate preference or that preference for nectar amino acids is condition or sex dependent (Alm et al., 1990; Erhardt, 1991; Mevi-Schütz and Erhardt, 2003; Mevi-Schütz et al., 2003). Collectively, these results suggest that, although widespread, preference for amino acid-containing nectars may not be broadly generalizable to species that vary in feeding habit or the degree to which adult diet affects life history traits (Boggs, 1997). Furthermore, demonstration of an existing preference may be masked in behavioral trials, either by previous experience or by aspects of an individual's current nutritional state/motivation (Takasu and Lewis, 1993; Cohen and Voet, 2002; Marsh et al., 2004; Toth et al., 2005; Akami et al., 2019).

In our 2-choice innate preference tests, naive *M. sexta* moths demonstrated a strong response to changing sucrose concentrations, with the PI approaching zero (no preference) in regular increments as the difference in sucrose concentration between experimental flowers also approached zero (25% versus 25% control treatment) in 5% decrements. While the addition of amino acids to these nectar solutions did not meet the criterion for statistical significance (*P*=0.06), there was a general trend for increased preference for nectar mimics containing amino acids (Fig. 3). Notably, this trend was seen even at low sucrose concentrations, and the PI approached zero (no preference between nectars) when sucrose concentrations remained unequal, before shifting negatively and indicating a preference for the amino acid-containing nectar at equal sucrose concentrations.

While this initial preference experiment detected a strong behavioral response to changing sucrose concentrations, the marginal result in relation to amino acids is not surprising. In this experiment, naive moths were expected to discriminate between nectars of variable quality in the first few minutes after foraging for the first time. Even in treatments containing only sucrose, there is substantial error and consumption of lower quality nectar solutions. Amino acids, while relevant to adult health and reproductive fitness in some circumstances (Mevi-Schütz and Erhardt, 2005), are not predicted to have a larger effect on nectar preference than sugar and caloric value. Furthermore, in this experiment none of the tested nectar solutions can be considered aversive or below an acceptance threshold, which limits the range of expected behavioral responses to more subtle differences in preference rather than acceptance versus rejection behaviors. Despite what can appear to be an instinctual ability to discriminate between and handle flowers while foraging, naive pollinators are frequently quite poor at exploiting floral resources prior to gaining experience (Laverty, 1994; Goyret and Raguso, 2006; Raine and Chittka, 2007). Our results may suggest that flower-naive hawkmoths do not exhibit subtle nectar preferences in their initial, exploratory foraging bouts.

In our subsequent learning experiments, in which *M. sexta* moths were given 3 days of foraging experience, the initial (yellow) color bias detected in first-choice measurements was maintained across all 3 days. However, the addition of amino acids to the experimental nectar or a 5% sucrose difference between nectars were equally effective in training moths away from their initial color bias (yellow) in favor of increasing visitation to the initially less preferred color (blue). One caveat to these experiments is that we did not conduct non-rewarding test trials to evaluate foraging choice in the absence of all reward or potential reward cues: the absence of nectar rewards alters behavior and dramatically reduces foraging in *M. sexta* to levels that are not sufficient to complete the appropriate test trials (Brandenburg et al., 2012). Instead, we used standardized artificial nectar and employed methods previously used to successfully evaluate learning in hawkmoths (see Materials and Methods, and Kelber, 1996) to reduce or eliminate the potential for alternative cues or modalities to interfere with learned associations between flower color and nectar composition. Separate from cues, experienced *M. sexta* demonstrated a preference for experimental nectars containing amino acids which was similar in magnitude to the response to a 5% sucrose difference between nectars. This approximate equivalence in which the presence or absence of amino acids produces an effect similar to, and statistically indistinguishable from, a 5% difference in sucrose concentration is remarkably similar to the trend suggested in the initial innate preference experiments (Fig. 3). Additionally, while different in mechanism, this trend is similar to the previously mentioned result (Rodríguez-Peña et al., 2013) in nectar-foraging bats, in which the presence of amino acids influences a foraging bat's ability or motivation to distinguish between nectars of different sugar content.

It is likely that this approximate equivalence varies across species and in relation to amino acid concentration, the neurophysiological mechanism of perception and the nutritional state of the pollinator. Irrespective of the exchange rate, however, these results suggest that in addition to enhancing perceived nectar quality in comparisons between solutions of equal sugar content, the addition of amino acids may be equally able to increase the perceived quality of a nectar that would otherwise be considered low quality when judged on caloric value or sucrose content alone. From the plant's perspective, this might suggest a potential strategy to enhance nectar quality while conserving photosynthate, as naturally occurring concentrations of nectar amino acids are typically orders of magnitude below those of nectar sugars (Petanidou et al., 2006; Nicolson and Thornburg, 2007). From the perspective of a foraging pollinator, this may have interesting implications as a nectar approaches its critical viscosity – the point at which increasing nectar sugar concentration and nectar viscosity impose diminishing returns on net energy intake rate (Kingsolver and Daniel, 1979; Harder, 1986; Josens and Farina, 2001). At this stage, the addition of nectar amino acids may enhance the perceived quality of a nectar beyond the profitability limits imposed by viscosity. Intriguingly, the addition of amino acids may affect nectar viscosity less than a direct increase in nectar sugar concentration (Heyneman, 1983). This dynamic could lead to conditions in which nectar enhancement via the addition of amino acids is preferential to a direct increase in sugar concentration, even in cases where the amino acids themselves may yield no additional nutritional benefit.

This study, while focusing on the foraging behavior of a large, well-studied insect pollinator, nevertheless was limited in ‘dimensional space’, with foraging experience, nectar sugar and amino acid content being the primary areas of our experimental focus. While these primary areas of focus involved degrees of preference for nectars of variable quality, our results also demonstrate learned avoidance of non-rewarding or aversive nectars (Fig. 5). These findings are consistent with previous studies involving selectively-bred or gene-silenced plants in which *M. sexta* moths exhibited reduced probing behavior or attenuated visitation to flowers with reduced nectar volumes or nectar containing defensive alkaloids (Kessler and Baldwin, 2007; Brandenburg et al., 2012; Kessler et al., 2015). Taken together, these studies describe a nectar-foraging pollinator that is capable of modifying its foraging decisions in response to non-sugar nectar compounds that either enhance or detract from its nutritional state.

When considered from a more natural, multidimensional context, we can imagine scenarios in which adult-acquired nectar amino acids might be of increased importance. For example, foraging preferences for nectar amino acids may be enhanced in reproductive female hawkmoths while provisioning maturing eggs. Female *M. sexta*, like some other adult feeding hawkmoths, mature the majority of their eggs after emerging from pupation (Sasaki and Riddiford, 1984; von Arx et al., 2013). Previously, the role of adult dietary amino acids in egg provisioning was thought to be quite limited; however, recent studies using more sensitive techniques have demonstrated that both essential and non-essential amino acids are regularly used to provision eggs and that the role of the adult diet increases over time during a female moth's reproductive life (O'Brien et al., 2002; Levin et al., 2017a). Similarly, in reproductive male *M. sexta*, adult nutrition plays an increasing role in the production of successive spermatophores (Levin et al., 2017b). Furthermore, in both sexes of *M. sexta*, adult dietary amino acids play a role in the maintenance of flight muscles and are also directly metabolized as fuel for flight (Levin et al., 2017a,b). Utilization of these adult dietary amino acids in a variety of physiological roles likely suggests that adult nutrition is involved in mediating several nutrition-related life history tradeoffs. For example, the use of dietary amino acids in maintaining flight muscles and as fuel for flight may modulate the strength of potential flight–reproduction tradeoffs, exemplified by wing-dimorphic insect species (Tanaka, 1993), but also demonstrated in some wing-monomorphic insects (Tigreros and Davidowitz, 2019). This dynamic may also be relevant in reproductive–immune system tradeoffs, as both systems can be limited by protein availability (Schwenke et al., 2016).

The adult *M. sexta* used in our experiments were young, virgin animals which were fed *ad libitum* during larval development. In their natural environment, hawkmoths are considered relatively long lived and are well known to forage and disperse pollen across long distances (Haber and Frankie, 1989; Skogen et al., 2019). Given the importance of adult dietary amino acids to hawkmoth survival, flight and reproduction, it is likely that older, reproductive animals or animals experiencing other competing nutritional demands would exhibit stronger preferences than those detected in these experiments. Considering our results as well as the life history and ecological context of *M. sexta*, it is reasonable to conclude that learning floral cues that are predictive of nectar composition beyond simply sugar concentration may provide foraging hawkmoths with an avenue to enhance survival and lifetime reproductive fitness.
